# Structures and stability of simple DNA repeats from bacteria

**DOI:** 10.1042/BCJ20190703

**Published:** 2020-01-22

**Authors:** Vaclav Brazda, Miroslav Fojta, Richard P. Bowater

**Affiliations:** 1 Institute of Biophysics of the Czech Academy of Sciences, Královopolská 135, 612 65 Brno, Czech Republic; 2 School of Biological Sciences, University of East Anglia, Norwich Research Park, Norwich NR4 7TJ, U.K.

**Keywords:** DNA metabolism, DNA structure, microsatellites, nucleic acids, repetitive DNA sequences

## Abstract

DNA is a fundamentally important molecule for all cellular organisms due to its biological role as the store of hereditary, genetic information. On the one hand, genomic DNA is very stable, both in chemical and biological contexts, and this assists its genetic functions. On the other hand, it is also a dynamic molecule, and constant changes in its structure and sequence drive many biological processes, including adaptation and evolution of organisms. DNA genomes contain significant amounts of repetitive sequences, which have divergent functions in the complex processes that involve DNA, including replication, recombination, repair, and transcription. Through their involvement in these processes, repetitive DNA sequences influence the genetic instability and evolution of DNA molecules and they are located non-randomly in all genomes. Mechanisms that influence such genetic instability have been studied in many organisms, including within human genomes where they are linked to various human diseases. Here, we review our understanding of short, simple DNA repeats across a diverse range of bacteria, comparing the prevalence of repetitive DNA sequences in different genomes. We describe the range of DNA structures that have been observed in such repeats, focusing on their propensity to form local, non-B-DNA structures. Finally, we discuss the biological significance of such unusual DNA structures and relate this to studies where the impacts of DNA metabolism on genetic stability are linked to human diseases. Overall, we show that simple DNA repeats in bacteria serve as excellent and tractable experimental models for biochemical studies of their cellular functions and influences.

## Simple DNA repeats

DNA molecules are the store of genetic information for all cellular organisms. The arrangements of individual bases in the DNA sequences of an organism, its genome, are specific to that organism, and elucidation of massive numbers of genome sequences have impacted on our understanding of the phylogenetic tree of life [1]. The organization of sequences in any genome is critical for its function and, from the earliest days of genome sequence analysis, it was recognized that natural DNA molecules contain a wide array of repeating sequences [2]. In fact, this was particularly important in many genomic studies because such sequences are challenging to obtain accurate data [3]. Repeat sequences of ∼1–6 base pairs (bp) in their unit structure are termed simple repeating sequences, due to their sequence being less complex (‘simpler’) than random sequences [4,5]. Such simple sequences are often called microsatellites and the term ‘short tandem repeats’ is also used frequently in the literature. Although most base sequences will be found within double-stranded DNA molecules, within this review we generally refer to sequences via a single strand, given in the 5′-3′ direction.

**Figure 1.**
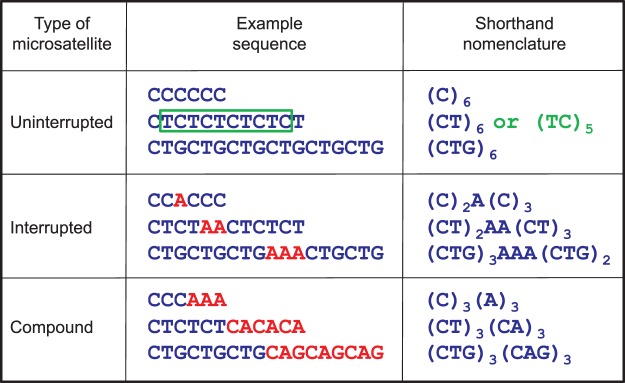
Nomenclature to illustrate variations of microsatellites repeats. Microsatellite sequences consist of up to six bases per repeat and examples are shown for microsatellite repeats consisting of one base (mononucleotide), two different bases (dinucleotide), and three different bases (trinucleotide). Note that shifting of the frame of the sequence highlights redundancy within each repeat, meaning that it covers multiple types of sequences — the green box highlights (TC) repeats within (CT) repeats. Classical examples of microsatellites consist of uninterrupted repeats of the same sequence. When one or more bases interrupt the repeat array (shown by the bases in red), the microsatellite is termed ‘interrupted’ (sometimes referred to as ‘imperfect’). Two types of repeat that neighbour each other are also found frequently in genomes, and are called ‘compound’ (or sometimes ‘composite’) microsatellites. Adapted from [4].

Simple repeating sequences can be distinguished by their sequence motif and base composition [4–7]. The various sequence motifs consist of different lengths of the repeat unit, such as mono-, di-, tri-, or tetranucleotide repeats, etc. For example, mononucleotide repeats are tracts of a single nucleotide in the sequence. Within repeating units there is some redundancy within DNA sequences e.g. (CT)*_n_* also contains (TC)*_m_*, where ‘*n*’ and ‘*m*’ refer to numbers of repeats — see Figure 1. (Depending on the sequences that flank the repeat, ‘*n*’ and ‘*m*’ may be equal, or they may differ by 1.) Importantly, DNA molecules have a directionality associated with them, with the 5′- and 3′-ends usually containing terminal phosphate and terminal hydroxyl groups, respectively [8]. Following the convention of writing sequences in a 5′-3′ direction and antiparallel arrangement of complementary chains in double-stranded DNA molecules, there are just two options for mononucleotide repeats (A/T or C/G base pairs) and four different types of dinucleotide repeats, (AT)*_n_*, (GT)*_n_*, (GA)*_n_*, and (GC)*_n_*. A similar analysis of trinucleotide repeats identifies ten different repeat sequences [9]. Classical examples of microsatellites consist of uninterrupted sequence of tandem repeats of the same motif (Figure 1). When one or more bases interrupt the repeat array, the microsatellite is termed ‘interrupted’ (also sometimes called ‘imperfect’). Juxtapositions of two types of repeat (called ‘compound’ or sometimes ‘composite’ microsatellites) also occur frequently in genomes (Figure 1).

**Figure 2.**
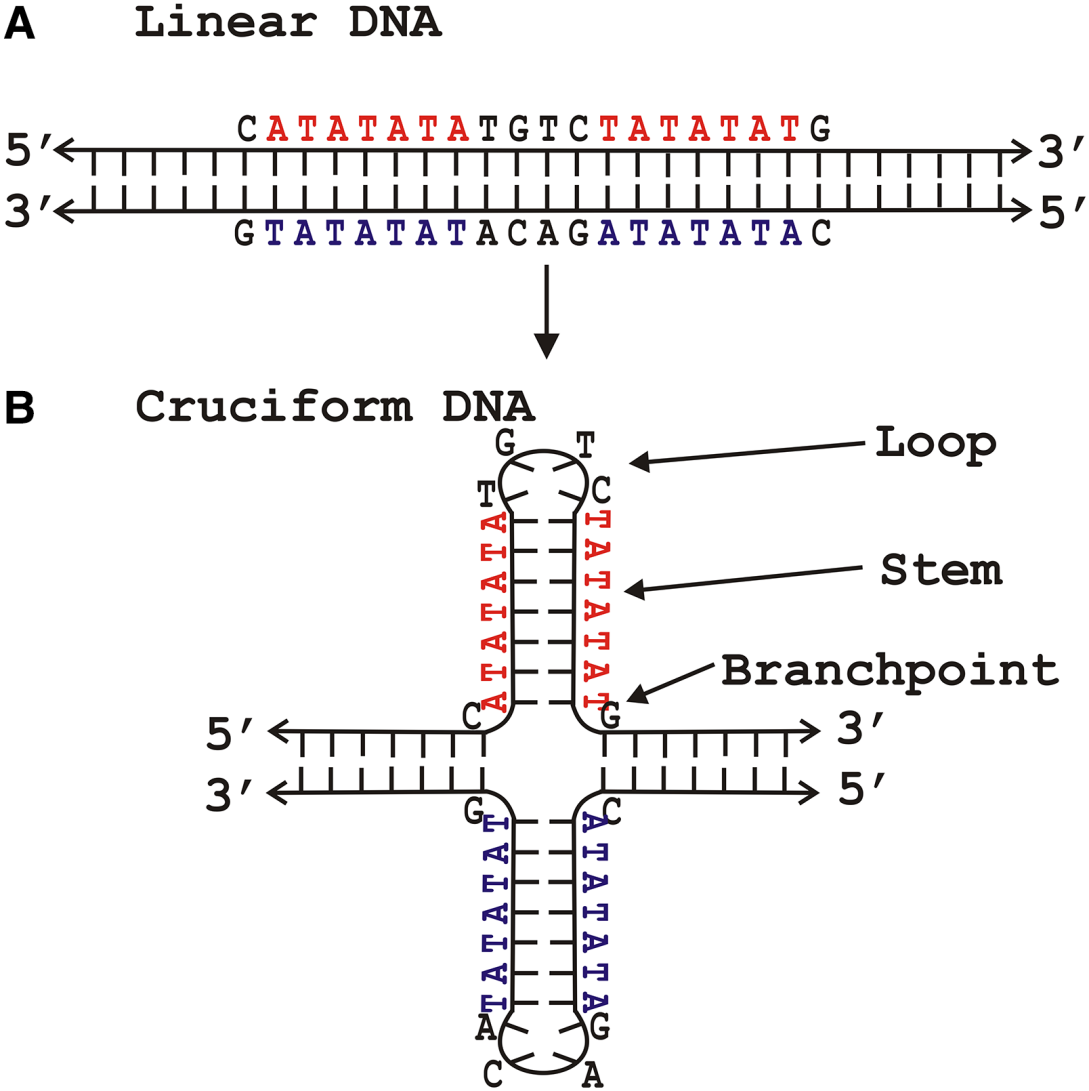
Inverted repeat DNA sequences can adopt different types of three-dimensional structure. ‘Inverted repeats’ are repetitive DNA elements where the 5′-3′ sequence of one strand is the same when the complementary strand is read in the 5′-3′ direction. The sequence shown is the inverted repeat from *E. coli K12* genome 3144772–3144797. (**A**) Such DNA sequences can exist in a regular double-stranded, antiparallel form. (**B**) Intra-strand base pairing within the inverted repeat allows the formation of a cruciform.

Some repetitive elements are referred to as ‘inverted repeats’ because the rules of complementary base pairing mean that their sequence is the same when the complementary strand is read in its 5′-3′ direction (Figure 2A) [10]. Since inverted repeats will occur on both strands at the specific location, they can adopt a specific structure referred to as a cruciform (Figure 2B) — see below for more details. Such sequences are targets for many architectural and regulatory proteins and their importance has been demonstrated for several basic biological processes. As we discuss below, such processes may be regulated by the formation of specific types of localized DNA structures at these sequences.

## Prevalence of DNA repeats in bacterial genomes

Advances in DNA sequencing technologies have generated massive numbers of genome sequences for prokaryotes due to their relatively small size and ease of experimental manipulation [1]. Most genome sequences are deposited in databases that make them publicly available. One such archive is the genome database at the National Center for Biotechnology Information (NCBI) and it contains DNA sequences from over two hundred thousand bacteria (206 445) as of 13/09/2019).

One of the first sequenced and best characterized bacterial genome is that of *Escherichia coli*, which contains a 4.6 million base pair genome with 4288 annotated protein-coding genes, seven ribosomal RNA operons, and 86 transfer RNA genes [11]. It is clear that there is a massive variation in phenotypes of bacteria, which is reflected in the huge variety of sizes and types of sequences found within their genomes. The vast majority of bacterial genomes are circular, consisting usually of large chromosomes and small plasmids. However, this is not always the case and there are notable examples of bacteria that harbour linear genomes, including some that are industrially important, such as *Streptomyces coelicolor* [12,13]. Indeed, there is vastly more evolutionary divergence among bacteria than is found among all other organisms on earth [1]. Many of the examples discussed in this review refer to *E. coli* because that system allows good correlation between bioinformatics and laboratory-based biological studies, but representative details from other organisms are discussed as appropriate.

All DNA genomes contain amounts of repetitive sequences that are larger than expected for random distribution of bases, but the percentage of repetitive sequences varies greatly across different organisms. For example, while the genome of *E. coli* contains only 0.7% of repeats in non-coding regions [11], at least 50% of the human genome is repetitive or repeat-derived [3]. As discussed in more detail below, through their involvement in DNA metabolism, repetitive DNA sequences have a dramatic influence on the genetic instability and evolution of genomes and organisms. These factors are some of the major forces that drive the increased prevalence of repeats within genomes compared with what would be expected if all bases were distributed randomly.

While simple DNA repeats are over-represented in the human genome and, generally, in eukaryotic genomes [14], in bacteria they are less common and are often subjected to negative selection [15]. However, significant differences in the amounts of simple DNA repeats exist, even among closely related species, as shown in mycoplasma [16]. An algorithm was developed to search specifically for tandem repeats [17]. Refinement of these approaches has developed computer-based analyses of microbial whole genome sequences that reveal overrepresentation of several simple DNA repeats. Such screening of the genome sequence of *E. coli* strain K12 identified thousands of tandem simple sequence repeat tracts, with motifs ranging from 1 to 6 nucleotides [18]. In addition to simple microsatellites, the repeats also consist of transposable genetic elements.

Comprehensive analyses of DNA sequence frequencies in various genomes have been published in the genome composition database (GCD) [19]. The genome-wide analysis of *E. coli* strain K12 already referred to shows a significant excess of mono- and trinucleotide repeats only [18]. The presence of the mononucleotide repeats is unequal for the two types and differs according to the GC contents of individual organisms [20]. For example, the GC content of *E. coli* K12 strain is 50.79%, but 93% of the mononucleotide repeats in its genome are formed by A (or T, its complement), both in open reading frames (ORFs) and in non-coding regions [18]. Similarly, the distribution of dinucleotide repeats in the genome of *E. coli* strain K12 is not random, with the (CG)*_n_* motif being very abundant in coding regions (49.1% of all dinucleotide repeats, compared with 17.3% expected).^1^
^1^The expected frequencies referred to here were determined by observing those in 10 computer-generated genomes constructed by random ordering of nucleotides according to their overall frequencies in the genome, with departures tested using parametric statistics. In non-coding regions, the (AT)*_n_* motif is over-represented relative to its expected value (24.4% compared with 17.9% expected), as is (CG)*_n_* (23.1% compared with 15.4% expected). Trinucleotide repeats are of particular interest to researchers because genetic instabilities within some of them are associated with a range of human diseases (see below). In *E. coli* strain K12 there is a significant excess of trinucleotide repeats, although their maximum observed number of repetitions is only five [18].

Similar analyses of repeats with larger unit lengths also showed that not all combinations are equally distributed in genomes. In *E. coli* strain K12 the maximum observed repeat length is four for tetranucleotide repeats, there are no pentanucleotide repeats and only three hexanucleotide repeats [18]. Furthermore, the frequencies of repeats with a specific motif of three and more bases were not distributed equally across all possible combinations. Most notably, 52 examples of tetranucleotide repeats, (TGGC)*_n_* (and its complement (GCCA)*_n_*) occurred 21 times in coding sequences. The finding that the *E. coli* genome is rich in (TGGC)*_n_* has been attributed to the activity of very short patch repair, which corrects T : G mismatches to C : G, thus increasing GC dinucleotide content in the genome [21].

The length and type of simple repeat sequences also vary significantly in different locations of genomes. For example, simple repeats that are rich in G bases on one strand (and C bases on the other) are often located at the ends of chromosomes. Known as telomeres, these repeats have been best characterized in the genomes of eukaryotes [22], but they also occur in some bacteria [12,13].

Analyses of short simple repeats among different strains of *E. coli* show that the number of repeats is polymorphic [23]. Determination of the size of repeat tracts can be used to identify different strains as long as care is taken to be aware of the potential for variable sizes to be identified in short repeats [24]. This approach can quickly diagnose the presence of different strains of bacteria, allowing identification of those that may be pathogenic, as demonstrated with *E. coli* [25,26], *Staphylococcus aureus* [27], *Mycobacterium leprae* [28], and many others [24].

## DNA structures formed by DNA repeats

**Figure 3.**
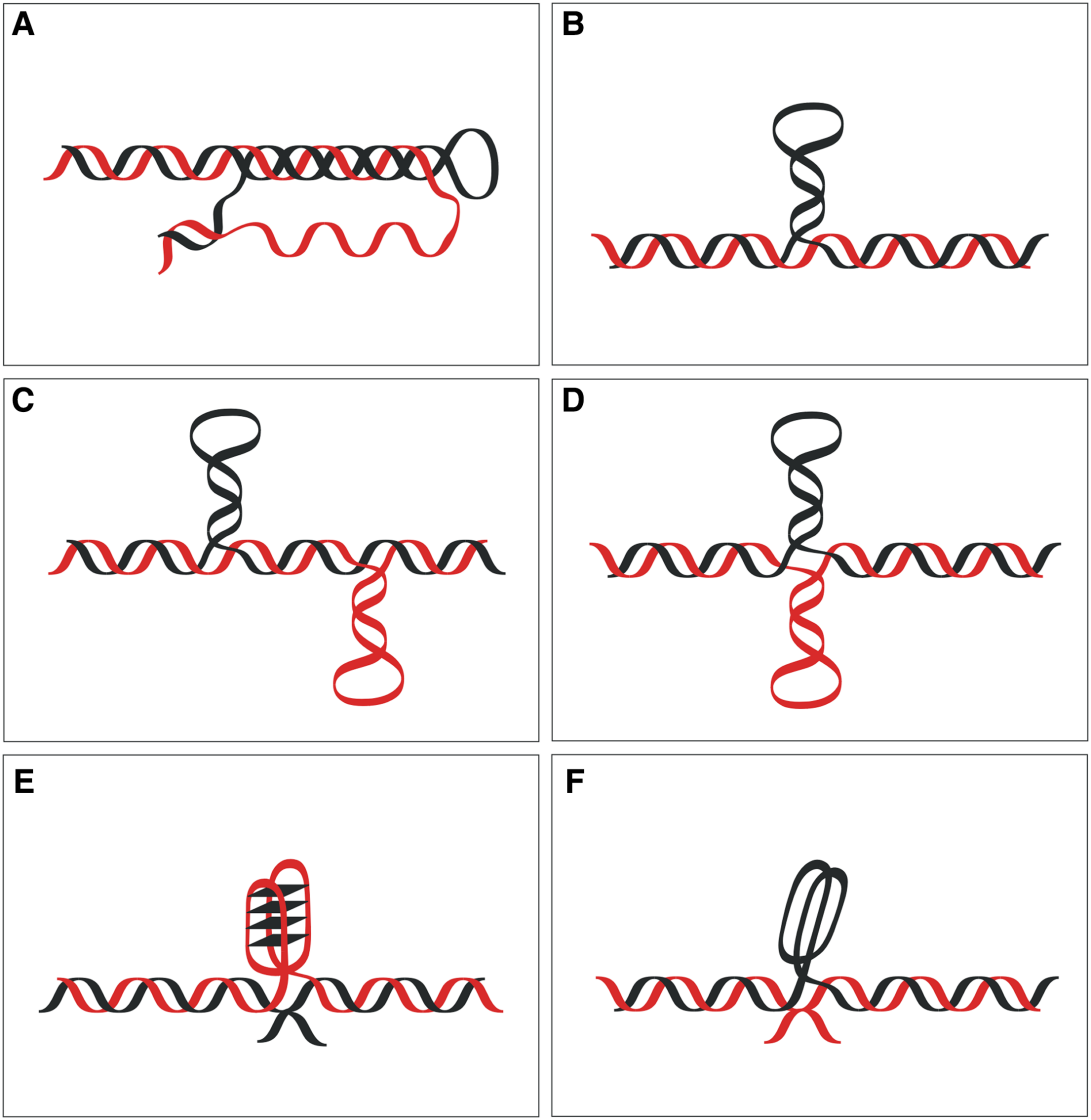
Ribbon scheme of localized non-B-DNA structures. (**A**) triplex; (**B**) hairpin; (**C**) slip-stranded DNA; (**D**) cruciform; (**E**) G-quadruplex; (**F**) i-motif. Black and red represent individual DNA strands, and G-quartets are highlighted by rhomboids.

DNA molecules, including those containing repetitive sequences, mostly form the two-stranded, right-handed helical B-form structure [8]. This structure maximizes the thermodynamic stability of the molecule and is crucial for fundamental biological processes that store, replicate, and transcribe genetic information. Nevertheless, various alternative (non-B) structures can also occur in DNA. These structures are usually characterized by the occurrence of single-stranded regions (loops) and/or sites of disrupted base pair stacking (junctions between continuous B-form DNA and the alternative structure). Since the disruption of hydrogen bonds and stacking interactions represents a loss of enthalpic contribution to the free energy of the molecule, any transition from B-form DNA to an alternative structure requires an input of energy. An alternative structure can be favoured if there are alterations to the sequence of one strand, for example when the complementary strand is absent or present in a sub-stoichiometric amount (as in the structure depicted in Figure 3B). However, some environmental (and cellular) conditions promote the formation of alternative structures due to their improved thermodynamic stability compared with B-form DNA under the given conditions. This type of situation occurs for some types of repetitive DNA sequences *in vitro*, with increasing evidence that such structures also exist within cells (see below). The types of the structure adopted by repetitive DNA sequences — and their thermodynamic stabilities — are influenced by the length and type of bases within the repeat. Furthermore, topological stress, which is inherent to the majority of DNA molecules inside cells, is another important factor that influences local DNA structures. Typically, DNAs in bacterial cells exist as negatively supercoiled molecules, which can lead to destabilization of right-handed, double-helical DNA [29,30]. In the presence of suitable nucleotide sequences, certain levels of negative superhelical stress can be locally absorbed via the transition from the B-form DNA to an open local structure. This can assist the formation of non-B-DNA structures, as shown *in vitro* for various types of repeats [31–34]. Evidence is particularly strong to show that higher levels of negative supercoiling increase the extent of cruciform formation in dinucleotide repeats. This has been confirmed for (AT)*_n_* sequences *in vitro* and in *E. coli* [29,35]. Variations in levels of DNA superhelicity naturally occurs *in vivo* in ‘active’ regions of the genome, where processes that involve unravelling of the DNA double helix take place, such as transcription, replication, and recombination.

**Figure 4.**
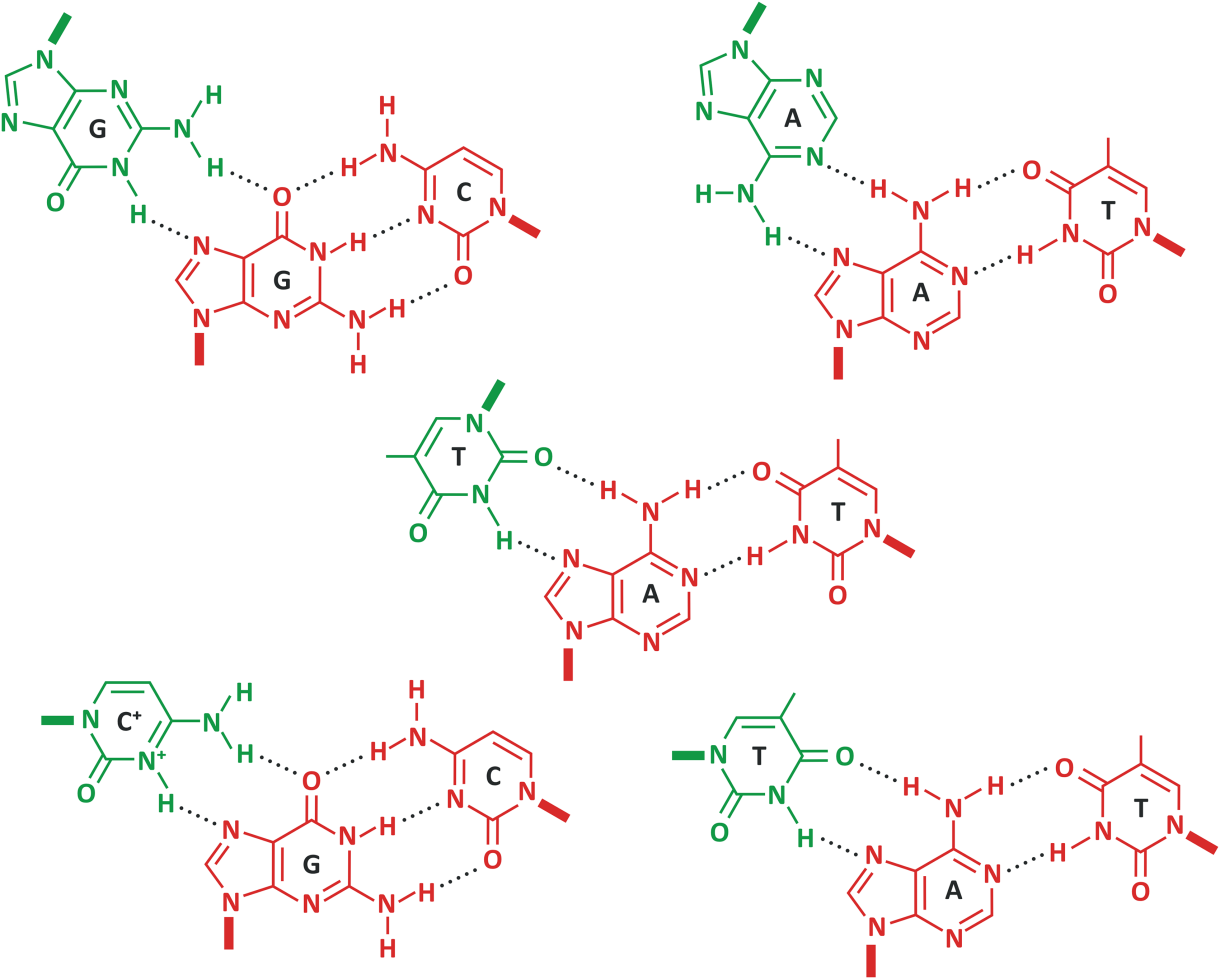
Watson–Crick and Hoogsteen hydrogen bonds in triplex DNA molecules. A variety of triplex structures are shown involving three separate bases. The most common sequences, both in intramolecular and intermolecular triplexes, include A•A–T, G•G–C, and T•A–T for R•R–Y type triplexes (bottom left), and C+•G–C and T•A–T for Y•R–Y type triplexes. Each triplex includes two bases that form hydrogen bonds following the standard (Watson–Crick) pattern (red), plus one additional base form basepair where the interactions are stabilized by Hoogsteen pairing (green) [66]. Note that in some cases the additional hydrogen bonds are stabilized by positive charges on a cytosine base and, thus, are favoured at low pH.

Due to complementary base pairing in double-stranded DNA, mononucleotide repeats are inherently homopurine on one strand and homopyrimidine on the other. While A tracts are prone to DNA bending [36], homopurine/homopyrimidine tracts, in general, are able to form triplex structures (Figure 3A). Mononucleotide repeats naturally possess mirror symmetry, which is a feature favouring triplex structures via the formation of Hoogsteen triads, as shown in Figure 4. Hoogsteen hydrogen bonding occurs between the purine-rich strand of the duplex DNA and either a pyrimidine-rich or a purine-rich third strand. Pyrimidine-rich third strand interactions are stabilized by Hoogsteen hydrogen bonds that are favoured at low pH, which facilitates the requirement for cytosine protonation required for its Hoogsteen pairing. In contrast, purine-rich third strand interactions form reverse-Hoogsteen hydrogen bonds, which do not require acidic pH and are stabilized by bivalent cations.

Mononucleotide repeats can also undergo strand slipping transitions, resulting in extrusion of a hairpin (Figure 3B) or a pair of hairpins that are separated from each other (Figure 3C). The proclivity to strand slipping is a common feature of simple repeats, playing a crucial role in their change in size during replication [31,37]. Conditions for good thermodynamic stability of hairpins have been well characterized *in vitro* for trinucleotide repeats such as (CGG)*_n_*, (CAG)*_n_*, and (CTG)*_n_*, even though these contain base mispairs or wobble pairs, such as T•T, A•A, or G•G [38,39].

For dinucleotide repeats the length observed in typical microsatellites varies from 5 to 50 repeats. Importantly, while all dinucleotide sequences are direct repeats, some are also inverted repeats (e.g. (AT)*_n_* and (CG)*_n_*), whereas others are not (e.g. (AG)*_n_* and (AC)*_n_*). This is significant because those that are inverted repeats are able to form cruciform structures (Figures 2 and 3D). At the same time, these sequences are composed of (purine–pyrimidine)*_n_* motifs that are capable of forming a segment of left-handed, Z-form, double helix under certain conditions [40].

Tandem repeats involving G*_n_* blocks and mononucleotide repeats consisting of G-tracts are able to form quadruplex structures (Figure 3E). Such structures are typically formed when four G nucleotides can be brought together in a planar arrangement to form guanine quartets involving Hoogsteen G–G pairing (see Figure 4) and are usually stabilized by the presence of monovalent cations in the middle of each G-quartet. Note that the presence of G-tracts on one strand means that C-tracts must be present on the complementary strand, and such sequences can adopt other non-B-DNA structures, such as the i-motif, which we discuss in more detail below.

A strikingly wide range of sequences have been demonstrated to form stable G-quadruplexes under different environmental conditions [37,41]. All of these sequences are not classically considered as simple DNA repeats, but G-quadruplexes can be formed by various types of short repeats of G bases within longer sequences. Some of the sequences that can form G-quadruplexes are simple microsatellite sequences, such as trinucleotide and hexanucleotide repeats [42,43]. Other sequences that are more complex in the base composition can also form G-quadruplexes, but they all contain G-tracts that are repeated with specific periodicities. Within any particular sequence that can form G-quadruplexes the bases that separate the G-tracts may be different in type and number and, thus, they represent a complicated type of interrupted repeat tract (see Figure 1). A wide array of sequences have been shown to form quadruplexes, but longer G-tracts and shorter interruptions form more stable G-quadruplexes, although the size of the loop also impacts on the type of folding seen in stable quadruplexes [44]. Importantly, the likelihood of G-quadruplexes forming in genomes varies dramatically in different locations of DNA molecules [45]. For example, simple repeats that are rich in G bases are often found at telomeric ends of chromosomes and there is significant evidence that such sequences form complexes of proteins specifically bound to four-stranded structures [46]. Telomeres have been best characterized in the genomes of eukaryotes, including humans, but they also occur in some bacteria [12,13,22].

Non-B-DNA structures are also able to form within sequences that would not typically be able to form significant levels of base pairing. For example, mononucleotide C*_n_* sequences and repeats with C*_n_* blocks are able to form hairpins (Figure 3B) and i-motif structures (Figure 3F) under conditions allowing the formation of hemi-protonated C^+^/C base pairs [47,48]. Following similar arguments presented above for G-quadruplexes, sequences that can form i-motifs are not all classically considered as simple DNA repeats. However, all of these sequences do contain C tracts that are repeated with specific periodicities and, thus, are relevant to topics discussed in this review. The i-motif structures require four C-rich strands containing bases, which can be formed from four distinct strands, two hairpins each carrying two cytosine stretches, or from a single strand with four cytosine stretches [49,50]. Recent observations have indicated that it is possible to achieve stable i-motifs at physiological pH without the use of crowding agents, if there are at least five cytosine bases per tract [48,51].

Trinucleotide repeat sequences also adopt many of the structures described above that are dependent on environmental conditions and type of sequences. For example, they can form slipped-stranded DNA and hairpins, but (CGG)*_n_* have been shown to form G-quadruplexes under specific conditions [52,53]. R-loops (Figure 3G) are another altered structure, which can be thermodynamically stable in (CAG)*_n_* and (GAA)*_n_* [54,55]. Major structures formed by (GAA)*_n_* are triplexes in which the third strand can be derived from either the pyrimidine strand or the purine strand [56,57]. One related structure that has particularly high thermodynamic stability in these sequences has been referred to as ‘sticky DNA’ because of the way it brings together multiples triplexes [58].

Thus, many molecular and biochemical studies demonstrate that simple repeating DNA sequences form a wide array of non-B-DNA structures *in vitro*. Whether such structures influence biological processes and consequences are questions that have been addressed in different cell types, including several bacteria, as we now discuss.

## Biochemical and cellular impacts of simple repeat sequences in bacteria

**Figure 5.**
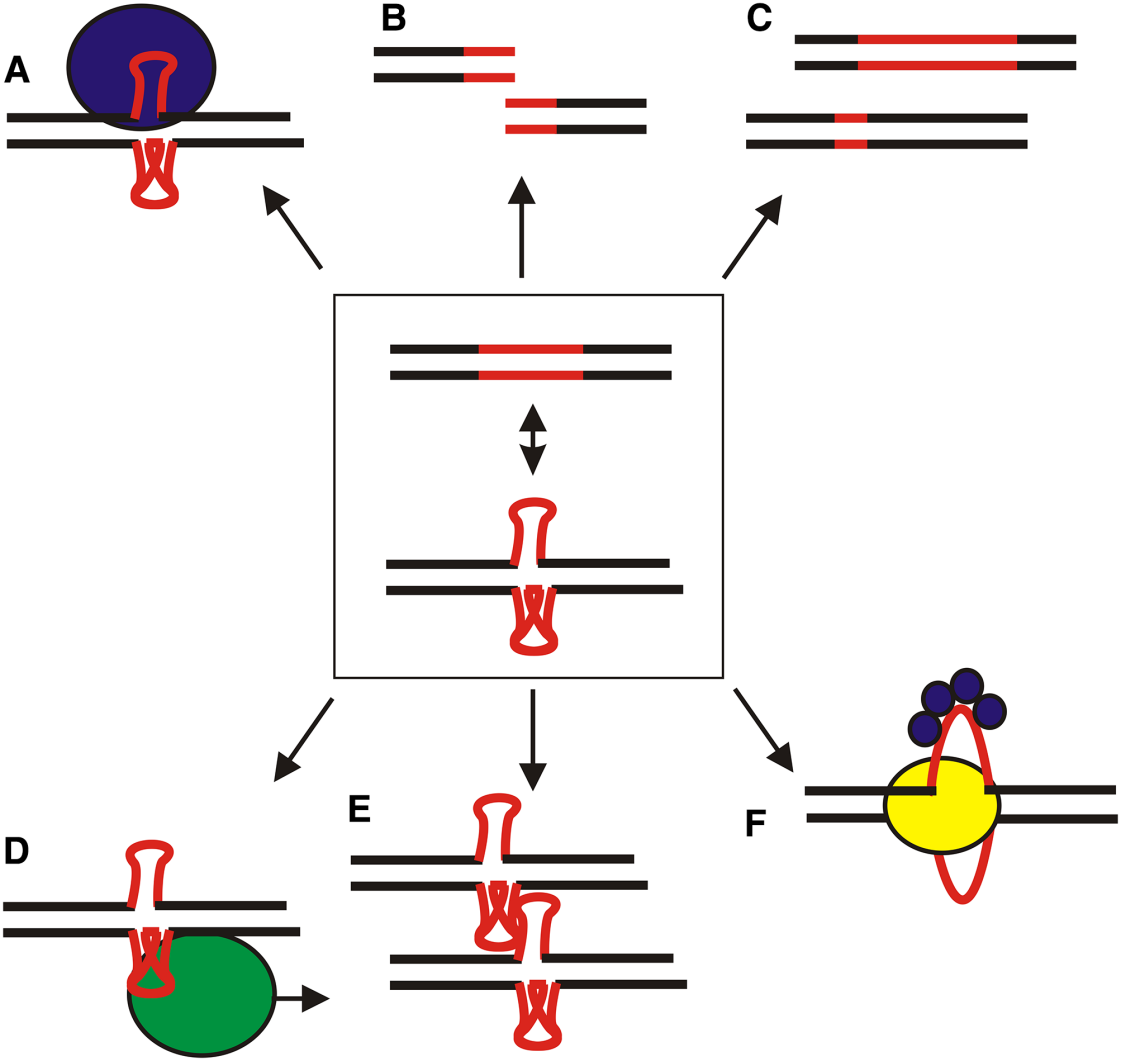
Suggested biological roles of simple DNA repeats. Central part: single DNA repeats (red) can form various local DNA structures (e.g. see Figure 3), which can participate in: (**A**) protein recognition; (**B**) genetic instability; (**C**) genome evolution; (**D**) regulation of transcription; (**E**) genome organization; (**F**) DNA replication. Colours highlight proteins with specificity for transcription (green), replication (yellow) or simply to the DNA structure or single-stranded DNA (blue).

Within the highly complex environment in cells, various local structures in long, genomic DNA molecules appear to serve as markers of the location of specific activities or functions. Examples of the types of cellular functions that they are involved in are highlighted in Figure 5. The biological relevance of these types of non-B-DNA motifs in recombination, replication, and the regulation of gene expression has long been proposed [59]. Furthermore, several studies have demonstrated the important role of non-B-DNA structures in the context of gene regulation in bacteria [30,60,61]. For example, cruciforms have been shown to be important for dynamic genome organization [62], and for replication of the circular molecules of genomes, plasmids, mitochondrial DNAs [63], and chloroplast DNAs [64]. Cruciforms are targets for many architectural and regulatory proteins [10] and their importance has been demonstrated for the regulation of transcription of some genes [65].

Three-stranded triplex structures can be formed in a range of simple repeats, and structures of many different types have been characterized [66]. Genomic loci containing motifs that can form triplexes are significantly more likely to undergo genome rearrangement compared with control sites, as demonstrated in certain Enterobacteria and Cyanobacteria species [67]. A systematic search of 5246 different bacterial plasmids and genomes for intra-strand triplex motifs was conducted and the results summarized in the ITxF database [66]. This database points to the importance of these types of sequences (and their potential to form non-B-DNA structures) in influencing the genetic stability of bacterial genomes.

**Figure 6.**
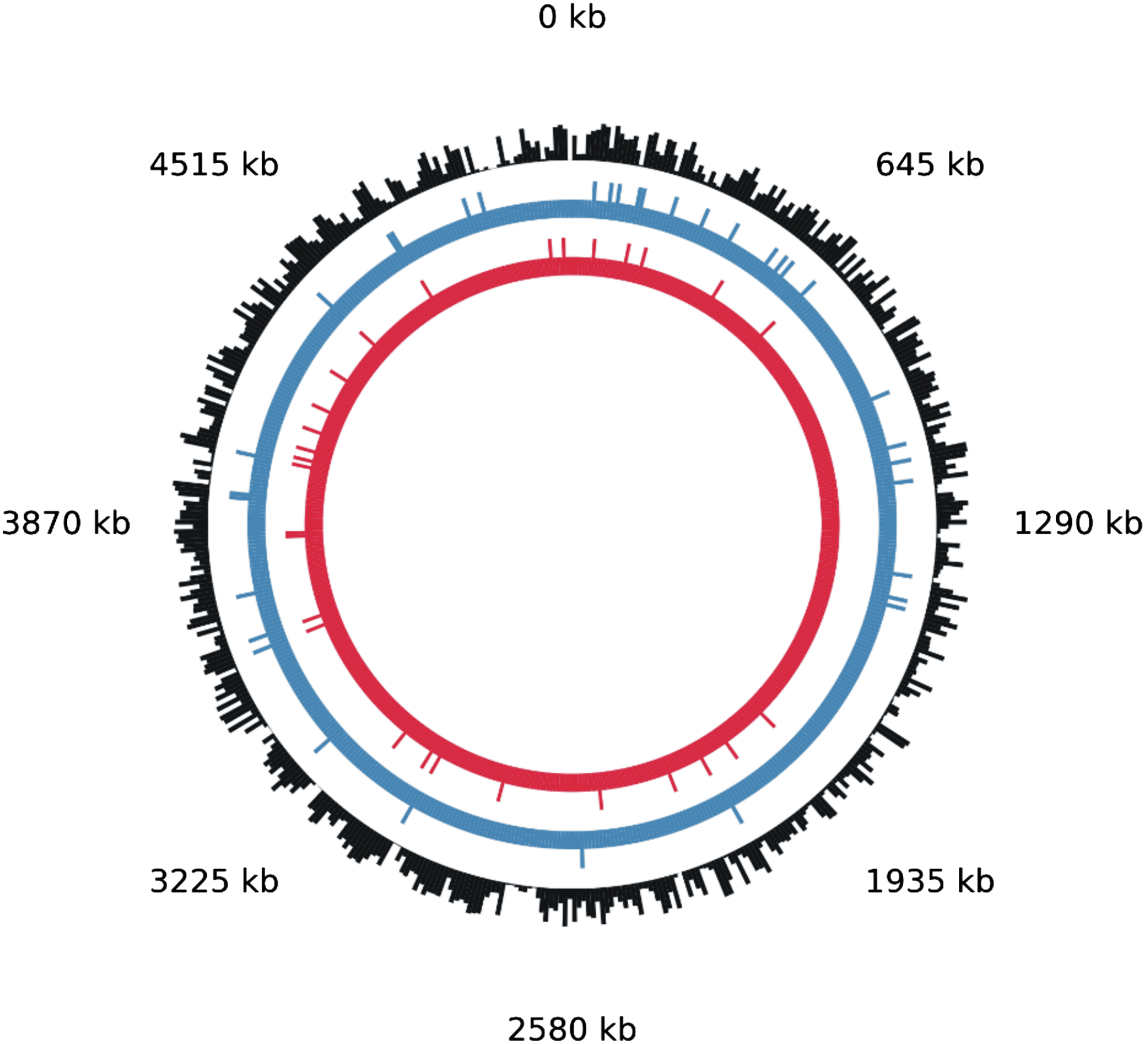
Potential quadruplex forming sequences are dispersed throughout the *Escherichia coli* genome. The presence of potential quadruplex forming sequences in *E. coli* 55989 was visualized by Quadbase [70,71]. The complete length of the genome is 5 154 862 bp, with a chromosomal CG content of 50.10% (outer black circle). The presence of quadruplex motifs is highlighted by the lines emanating from the inner circles: the ‘+ strand’ has 37 (middle blue circle), the ‘− strand’ has 28 (inner red circle).

Several bioinformatics tools have been developed to identify potential quadruplex sequences in genomes, such as QGRS Mapper [68] and G4Hunter [69]. In another example, the ProQuad database developed simple rules for G-quadruplex forming patterns and used them to assess the occurrence of repeating G-tracts and their association with different genomic regions. This initially identified potential quadruplex sequences within the genomes of 146 bacterial species [70], and an updated database, QuadBase2, mined motifs across genes and their promoter sequences in 1719 prokaryotes [71]. This database can be used to identify the number and location of repeats within large genome sequences. As an example, we use this to identify potential quadruplex forming sequences in the genome of *E. coli* K12 strain, highlighting 69 sequences, 37 in the plus strand and 28 in the minus strand (Figure 6). A separate genome-wide analysis of 18 microbes indicated enrichment of G-quadruplex DNA motifs in putative promoters, with detailed analysis in *E. coli* suggesting a global role for them in ‘turning-on’ transcription during certain growth phases [72]. Along with *in vitro* data that demonstrates quadruplexes are bound by some proteins [46,73], these findings point towards physiological functions for G-quadruplexes. In this respect, it is significant that genomes with high G + C content are more able to form four-stranded structures with relatively high thermodynamic stability [37,74]. There is increasing evidence that these types of structures provide opportunities to regulate DNA metabolism in bacteria [51,75–77]. The genome of the bacterium *Paracoccus denitrificans* PD1222 has a relatively high G + C content (∼67%) and a range of biophysical, molecular, and microbiological studies show that targeting of four-stranded structures can be controlled under cellular conditions, allowing regulation of expression of some genes [48,78–81].

Scientific interest in the genetic stability of simple DNA repeats took on much wider significance when it was recognized that length changes within them are linked to human diseases and disorders. In the 1990s, genetic instability of microsatellites was identified as a useful diagnostic tool for some types of cancer and is associated with some hereditary neurological disorders in humans [54,82–84]. Much effort has been put into analyzing cellular mechanisms that lead to genetic instabilities of trinucleotide repeats, aiming to understand why some are more prevalent in human disorders, the most common of which are CAG, CTG, CGG, and GAA. Recent molecular studies have confirmed that other simple repeats are also important for human diseases [54,58]. These links have driven many studies that focus on DNA repeats in bacteria where it is often more tractable to conduct genetic analyses.

Different models have been proposed to explain genetic instabilities observed in simple repeats. Many of them involve DNA synthesis, including DNA replication, and various types of DNA repair and recombination [7,31,33,82,84–86]. Extensive experiments using *E. coli* confirmed that length changes in plasmid-based DNA trinucleotide repeats are affected by replication. The observations are consistent with known biochemical properties of replication forks and lead to suggestions that the sequence within the repeat influences the thermodynamic stability of unusual structures in the DNA [31,33,84,86,87]. Other processes acting on DNA can impact on mechanisms by which DNA synthesis influences the genetic stability of simple repeats in *E. coli*. For example, transcription of DNA mononucleotide repeats blocked their subsequent replication [88], and transcription into trinucleotide repeats in plasmids influenced the frequency of deletions to the repeat [89–91]. These experiments highlight that interactions between different processes acting on DNA combine to influence their genetic instability. Interactions may be particularly relevant for processes that use similar proteins, such as DNA polymerases in DNA replication and repair.

The link to DNA repair systems has intriguing roles in relation to genetic instabilities of simple DNA repeats because some of them recognize any aspect of genome structure that is different from the standard base pairs and double helix, including non-B-DNA structures [33,74,92]. All cells contain proteins that recognize and repair such genome alterations, protecting genomic integrity by different pathways, which include mismatch repair (MMR), nucleotide and base excision repair, and the repair of double- and single-strand breaks [83,93,94]. Generally, the DNA repair pathways and their proteins are well conserved, which means that there is much to be gained from studies of these systems in simpler experimental models, such as bacteria [95–97]. As described below, experiments using bacteria, particularly different strains of mycobacteria, have been very useful for understanding how DNA repair systems influence the genetic stability of simple DNA repeats.

An important physiological role for some DNA repair pathways is to prevent significant changes to the type and number of bases within the genome. However, the genetic instabilities observed within DNA repeats indicate that modifications to the size of the genome are not always repaired. Possibly, cells may not be able to repair some types of length changes to repeats due to non-recognition of certain structures or inaccessibility of DNA processed by some events. Alternatively, mutations in repair proteins may induce length alterations to repeats. Numerous studies show that the impact of DNA repair pathways on repeat tract stability is complex [84,85]. Importantly, some non-B-DNA structures are identified as modifications to be removed, at least in some contexts or under certain conditions.

MMR and nucleotide excision repair (NER) are fundamental cellular systems involved in maintaining genomic integrity [82,83,85,93,94]. MMR is able to detect and replace mismatched base pairs that are introduced during inaccurate DNA synthesis. Without such repair, these mismatched base pairs are a source of mutations within genomes. Upon inactivation of MMR, increased heterogeneities have been observed at simple repetitive DNA (e.g. mono- and dinucleotides) in bacteria [82,87], suggesting that the genetic stability of simple repeats indicates the increased rate of mutation throughout the whole genome. Due to this phenomenon, such deficiencies within DNA repair systems have been termed the ‘mutator phenotype’ [98]. Generally, NER systems recognize a wide range of lesions and damage due to distortion of the DNA double helix, and unusual DNA structures that could form in repeat tracts are likely to be activators of NER [83,93,94]. Studies in *E. coli* observed that their constituent NER proteins influenced the genetic stability of long plasmid-based DNA trinucleotide repeats in a complex fashion [33,82,87]. Associations between defective MMR and NER and elevated microsatellite instability are linked to some human diseases, and are particularly strong for hereditary nonpolyposis cancer.

In contrast with their usual cellular functions, the excision repair systems can enhance the genetic instabilities of DNA repeats since they provide opportunities for non-B-DNA structures to form on single-stranded regions that are presented as the damage is excised from the DNA helix. Therefore, the repair processes themselves can lead to further consequences, such as addition or deletion of bases, which would be observed as genetic instability [82,85,93,94]. Furthermore, abundant evidence demonstrates that unusual DNA structures may be recognized as ‘damaged DNA’ by DNA repair systems, sometimes leading to the deletion of the sequence [92,99,100]. To reduce such potential problems, cells also take advantage of enzymatic processes to dissolve unusual DNA structures, such as DNA helicases [101]. For example, the RecQ helicases are capable of unwinding G-quadruplex DNA and this family of enzymes is conserved and is essential for genomic stability in organisms from *E. coli* to humans [102,103].

Genetic instabilities within mono- and dinucleotide repeats increase for longer runs of consecutive repeats and, therefore, are decreased by interruptions in the repeat sequence [87]. These observations are consistent with the hypothesis that slipped-strand mispairing during DNA synthesis generates misaligned intermediates. Such parameters are intrinsic to the DNA repeat, but flanking sequences also influence the genetic stability of simple repeat sequences. These observations suggest that many aspects of DNA metabolism affect the genetic stability of all microsatellite sequences.

Through their effects on DNA metabolism, repetitive DNA sequences have a dramatic influence on the genetic instability and evolution of genomes and organisms. The high levels of genetic instability of repetitive DNA sequences may act to promote the evolution of genomic sequences [84,104]. It has been suggested that length changes to simple repeats can normally be tolerated because they do not have dramatic consequences for the organism in question and that deleterious consequences occur only at extreme length changes [104,105], as described for the trinucleotide repeat diseases. However, it is clear that simple DNA repeats in bacteria represent hypermutable loci associated with reversible changes in the number of repeats [2,106]. Variability of the length of simple DNA repeats can lead to the increased antigenic variance of the pathogen population [107]. Such length changes have been clearly demonstrated in bacteria, where this property means that simple DNA repeats can act as prerequisites for bacterial phase variation and adaptation, providing clear evidence that length variations to repeat tracts are used as a means of modulating gene expression. For example, in some bacteria, such as *Haemophilus influenzae*, the susceptibility of microsatellites to reversible length changes is used to control specific genes that allow environmental adaptation [104,108]. Thus, the hypermutable repeat sequence allows the bacterium to respond swiftly to changes in environmental conditions and adapt to different situations [104,109]. Such variability in repeat tracts can even impact on the virulence of some bacteria, as seen in *H. influenzae* and *Neisseria meningitides* [110,111]. Variation in the overall size of the repetitive domains was detected even among bacteria sub-cultured from a single colony, highlighting that the altered size of the repeat was intrinsic to the sequence.

## Conclusions

From the earliest studies of natural DNAs, it became clear that repetitive DNA sequences are common, leading to expectations that there must be biological reasons to explain this. The advent of large numbers of genome sequences has reinforced these observations, but biologists continue to assess the full biological significance of repetitive regions of genomes. Different aspects of DNA metabolism influence genetic instabilities within these sequences, and many of the studies that have improved knowledge have originated in bacteria, where the experiments are most tractable. An important corollary of the results from such studies is that many of the biochemical pathways are found in all organisms, meaning that many of the conclusions are relevant to all organisms.

Genetic instabilities of simple repeats may be mediated by many biochemical processes, including DNA replication-based slipped-strand mispairing, small slipped-register DNA synthesis, tandem duplications, and gene conversion-recombination processes. These processes may occur independently or in concert with each other and/or other DNA metabolic processes such as MMR, NER, DNA polymerase proofreading, SOS repair, and transcription. It is also clear that the structural properties of the simple repeats (hairpin loop formation, slipped structures, triplexes, etc.) play a consequential role in their genetic instabilities. The involvement of unusual DNA structures may occur because they are inherent within simple repeats inside cells, or because enzymes manipulating DNA may promote their formation. Either way, the presence of unusual structures within simple repeats is likely to influence the interaction of the DNA with proteins, which, in turn, facilitates the genetic instability of simple repeats.

Rapid progress in obtaining and interpreting genome information will continue to extend knowledge about the genetic variations that exist for simple repeating DNA sequences across all organisms. In this review, we have summarized the current understanding obtained from biochemical and cellular studies of such repeat sequences in bacteria. A combination of these different experiments in bacteria will shed further insight into the biological impacts of simple DNA repeats, including enhancement in understanding their roles in bacterial metabolism (with possible impact in the treatment of bacterial pathogens) as well as in a range of human diseases.

## Abbreviations

bp: base pairs
CD: circular dichroism
DNA: deoxyribonucleic acid
GCD: genome composition database
MMR: mismatch repair
NCBI: National Center for Biotechnology Information
NER: nucleotide excision repair
NMR: nuclear magnetic resonance
ORF: open reading frame

## Open Access

Open access for this article was enabled by the participation of University of East Anglia in an all-inclusive *Read & Publish* pilot with Portland Press and the Biochemical Society under a transformative agreement with Jisc.

## References

[1] Castelle C.J. and Banfield J.F. (2018) Major new microbial groups expand diversity and alter our understanding of the tree of life. Cell 172, 1181–1197 10.1016/j.cell.2018.02.016 29522741

[2] Kashi Y. and King D.G. (2006) Simple sequence repeats as advantageous mutators in evolution. Trends Genet. 22, 253–259 10.1016/j.tig.2006.03.005 16567018

[3] Treangen T.J. and Salzberg S.L. (2011) Repetitive DNA and next-generation sequencing: computational challenges and solutions. Nat. Rev. Genet. 13, 36–46 10.1038/nrg3117 22124482 PMC3324860

[4] Schlötterer C. (2016) Simple repeats. In eLS, John Wiley & Sons, Ltd (Ed.) 10.1002/9780470015902.a0005066.pub2

[5] Mrazek J., Guo X. and Shah A. (2007) Simple sequence repeats in prokaryotic genomes. Proc. Natl Acad. Sci. U.S.A. 104, 8472–8477 10.1073/pnas.0702412104 17485665 PMC1895974

[6] van Belkum A., Scherer S., van Alphen L. and Verbrugh H. (1998) Short-sequence DNA repeats in prokaryotic genomes. Microbiol. Mol. Biol. Rev. 62, 275–293 10.1128/MMBR.62.2.275-293.1998 9618442 PMC98915

[7] Neil A.J., Kim J.C. and Mirkin S.M. (2017) Precarious maintenance of simple DNA repeats in eukaryotes. Bioessays 39, 1700077 10.1002/bies.201700077 PMC5577815 28703879

[8] Bowater R.P. and Waller Z.A. (2014) DNA structure. In eLS, John Wiley & Sons, Ltd (Ed.) 10.1002/9780470015902.a0006002.pub2

[9] Ohshima K., Kang S., Larson J.E. and Wells R.D. (1996) Cloning, characterisation, and properties of seven triplet repeat DNA sequences. J. Biol. Chem. 271, 16773–16783 10.1074/jbc.271.28.16773 8663377

[10] Brazda V., Laister R.C., Jagelska E.B. and Arrowsmith C. (2011) Cruciform structures are a common DNA feature important for regulating biological processes. BMC Mol. Biol. 12, 33 10.1186/1471-2199-12-33 21816114 PMC3176155

[11] Blattner F.R., Plunkett I., Bloch G., Perna C.A., Burland N.T., Riley V., (1997) The complete genome sequence of *Escherichia coli* K-12. Science 277, 1453–1462 10.1126/science.277.5331.1453 9278503

[12] Chen C.W., Huang C.H., Lee H.H., Tsai H.H. and Kirby R. (2002) Once the circle has been broken: dynamics and evolution of Streptomyces chromosomes. Trends Genet. 18, 522–529 10.1016/S0168-9525(02)02752-X 12350342

[13] Hopwood D.A. (2006) Soil to genomics: the streptomyces chromosome. Ann. Rev. Genet. 40, 1–23 10.1146/annurev.genet.40.110405.090639 16761950

[14] Toth G., Gaspari Z. and Jurka J. (2000) Microsatellites in different eukaryotic genomes: survey and analysis. Genome Res. 10, 967–981 10.1101/gr.10.7.967 10899146 PMC310925

[15] Field D. and Wills C. (1998) Abundant microsatellite polymorphism in *Saccharomyces cerevisiae*, and the different distributions of microsatellites in eight prokaryotes and *S. cerevisiae*, result from strong mutation pressures and a variety of selective forces. Proc. Natl Acad. Sci. U.S.A. 95, 1647–1652 10.1073/pnas.95.4.1647 9465070 PMC19132

[16] Mrazek J. (2006) Analysis of distribution indicates diverse functions of simple sequence repeats in Mycoplasma genomes. Mol. Biol. Evol. 23, 1370–1385 10.1093/molbev/msk023 16618962

[17] Benson G. (1999) Tandem repeats finder: a program to analyze DNA sequences. Nucleic Acids Res. 27, 573–580 10.1093/nar/27.2.573 9862982 PMC148217

[18] Gur-Arie R., Cohen C.J., Eitan Y., Shelef L., Hallerman E.M. and Kashi Y. (2000) Simple sequence repeats in *Escherichia coli*: abundance, distribution, composition, and polymorphism. Genome Res. 10, 62–71 PMID: 10645951 PMC310497

[19] Kryukov K., Sumiyama K., Ikeo K., Gojobori T. and Saitou N. (2012) A new database (GCD) on genome composition for eukaryote and prokaryote genome sequences and their initial analyses. Genome Biol. Evol. 4, 501–512 10.1093/gbe/evs026 22417913 PMC3342873

[20] Wu H., Zhang Z., Hu S. and Yu J. (2012) On the molecular mechanism of GC content variation among eubacterial genomes. Biol. Direct 7, 2 10.1186/1745-6150-7-2 22230424 PMC3274465

[21] Bhagwat A.S. and Lieb M. (2002) Cooperation and competition in mismatch repair: very short-patch repair and methyl-directed mismatch repair in *Escherichia coli*. Mol. Microbiol. 44, 1421–1428 10.1046/j.1365-2958.2002.02989.x 12067333

[22] Aksenova A.Y. and Mirkin S.M. (2019) At the beginning of the end and in the middle of the beginning: structure and maintenance of telomeric DNA repeats and interstitial telomeric sequences. Genes 10, E118 10.3390/genes10020118 30764567 PMC6410037

[23] Metzgar D., Thomas E., Davis C., Field D. and Wills C. (2001) The microsatellites of *Escherichia coli*: rapidly evolving repetitive DNAs in a non-pathogenic prokaryote. Mol. Microbiol. 39, 183–190 10.1046/j.1365-2958.2001.02245.x 11123700

[24] van Belkum A. (2007) Tracing isolates of bacterial species by multilocus variable number of tandem repeat analysis (MLVA). FEMS Immunol. Med. Microbiol. 49, 22–27 10.1111/j.1574-695X.2006.00173.x 17266711

[25] Dyet K.H., Robertson I., Turbitt E. and Carter P.E. (2011) Characterization of *Escherichia coli* O157:H7 in New Zealand using multiple-locus variable-number tandem-repeat analysis. Epidemiol. Infect 139, 464–471 10.1017/S0950268810001068 20478087

[26] Byrne L., Elson R., Dallman T.J., Perry N., Ashton P., Wain J. et al. (2014) Evaluating the use of multilocus variable number tandem repeat analysis of Shiga toxin-producing *Escherichia coli* O157 as a routine public health tool in England. PLoS One 9, e85901 10.1371/journal.pone.0085901 24465775 PMC3895024

[27] Melles D.C., Schouls L., Francois P., Herzig S., Verbrugh H.A., van Belkum A. et al. (2009) High-throughput typing of *Staphylococcus aureus* by amplified fragment length polymorphism (AFLP) or multi-locus variable number of tandem repeat analysis (MLVA) reveals consistent strain relatedness. Eur. J. Clin. Microbiol. Infect. Dis. 28, 39–45 10.1007/s10096-008-0585-4 18663501

[28] Mohanty P.S., Bansal A.K., Naaz F., Arora M., Gupta U.D., Gupta P. et al. (2019) Multiple strain infection of *Mycobacterium leprae* in a family having 4 patients: a study employing short tandem repeats. PLoS One 14, e0214051 10.1371/journal.pone.0214051 30947261 PMC6449029

[29] Bowater R.P., Chen D. and Lilley D.M.J. (1994) Elevated unconstrained supercoiling of plasmid DNA generated by transcription and translation of the tetracycline resistance gene in eubacteria. Biochemistry 33, 9266–9275 10.1021/bi00197a030 8049227

[30] Hatfield G.W. and Benham C.J. (2002) DNA topology-mediated control of global gene expression in *Escherichia coli*. Annu. Rev. Genet. 36, 175–203 10.1146/annurev.genet.36.032902.111815 12429691

[31] Pearson C.E., Edamura K.N. and Cleary J.D. (2005) Repeat instability: mechanisms of dynamic mutations. Nat. Rev. Genet. 6, 729–742 10.1038/nrg1689 16205713

[32] Mirkin S.M. (2006) DNA structures, repeat expansions and human hereditary disorders. Curr. Opin. Struct. Biol. 16, 351–358 10.1016/j.sbi.2006.05.004 16713248

[33] Wells R.D. (2007) Non-B DNA conformations, mutagenesis and disease. Trends Biochem. Sci. 32, 271–278 10.1016/j.tibs.2007.04.003 17493823

[34] Sjakste T., Paramonova N. and Sjakste N. (2016) Structural and functional significance of microsatellites. Biopolym. Cell 32, 334–346 10.7124/bc.000930

[35] McClellan J.A., Boublikova P., Palecek E. and Lilley D.M.J. (1990) Superhelical torsion in cellular DNA responds directly to environmental and genetic factors. Proc. Natl Acad. Sci. U.S.A. 87, 8373–8377 10.1073/pnas.87.21.8373 2172986 PMC54958

[36] Gimenes F., Takeda K.I., Fiorini A., Gouveia F.S. and Fernandez M.A. (2008) Intrinsically bent DNA in replication origins and gene promoters. Genet. Mol. Res 7, 549–558 10.4238/vol7-2gmr461 18752180

[37] Murat P. and Balasubramanian S. (2014) Existence and consequences of G-quadruplex structures in DNA. Curr. Opin. Genet. Dev. 25, 22–29 10.1016/j.gde.2013.10.012 24584093

[38] Mitas M. (1997) Trinucleotide repeats associated with human disease. Nucleic Acids Res. 25, 2245–2254 10.1093/nar/25.12.2245 9171073 PMC146772

[39] Kiliszek A. and Rypniewski W. (2014) Structural studies of CNG repeats. Nucleic Acids Res. 42, 8189–8199 10.1093/nar/gku536 24939898 PMC4117766

[40] Wang G. and Vasquez K.M. (2007) Z-DNA, an active element in the genome. Front Biosci. 12, 4424–4438 10.2741/2399 17485386

[41] Bochman M.L., Paeschke K. and Zakian V.A. (2012) DNA secondary structures: stability and function of G-quadruplex structures. Nat. Rev. Genet. 13, 770–780 10.1038/nrg3296 23032257 PMC3725559

[42] Malgowska M., Gudanis D., Kierzek R., Wyszko E., Gabelica V. and Gdaniec Z. (2014) Distinctive structural motifs of RNA G-quadruplexes composed of AGG, CGG and UGG trinucleotide repeats. Nucleic Acids Res. 42, 10196–101207 10.1093/nar/gku710 25081212 PMC4150804

[43] Zamiri B., Mirceta M., Bomsztyk K., Macgregor R.B. Jr. and Pearson C.E. (2015) Quadruplex formation by both G-rich and C-rich DNA strands of the C9orf72 (GGGGCC)8*(GGCCCC)8 repeat: effect of CpG methylation. Nucleic Acids Res. 43, 10055–10064 10.1093/nar/gkv1008 26432832 PMC4787773

[44] Cheng M., Cheng Y., Hao J., Jia G., Zhou J., Mergny J.L. et al. (2018) Loop permutation affects the topology and stability of G-quadruplexes. Nucleic Acids Res. 46, 9264–9275 10.1093/nar/gky757 30184167 PMC6182180

[45] Bartas M., Cutova M., Brazda V., Kaura P., Stastny J., Kolomaznik J., et al. (2019) The presence and localization of G-quadruplex forming sequences in the domain of bacteria. Molecules 24, 1711 10.3390/molecules24091711 PMC6539912 31052562

[46] McRae E.K.S., Booy E.P., Padilla-Meier G.P. and McKenna S.A. (2017) On characterizing the interactions between proteins and guanine quadruplex structures of nucleic acids. J. Nucleic Acids 2017, 9675348 10.1155/2017/9675348 29250441 PMC5700478

[47] Qiu J., Liu J., Chen S., Ou T.M., Tan J.H., Gu L.Q. et al. (2015) Role of Hairpin-Quadruplex DNA secondary structural conversion in the promoter of hnRNP K in gene transcriptional regulation. Org. Lett. 17, 4584–4587 10.1021/acs.orglett.5b02310 26332732

[48] Abdelhamid M.A., Fabian L., MacDonald C.J., Cheesman M.R., Gates A.J. and Waller Z.A. (2018) Redox-dependent control of i-Motif DNA structure using copper cations. Nucleic Acids Res. 46, 5886–5893 10.1093/nar/gky390 29800233 PMC6159522

[49] Dembska A., Bielecka P. and Juskowiak B. (2017) pH-Sensing fluorescence oligonucleotide probes based on an i-motif scaffold: a review. Anal. Methods 9, 6092–6106 10.1039/C7AY01942D

[50] Abou Assi H., Garavis M., Gonzalez C. and Damha M.J. (2018) i-Motif DNA: structural features and significance to cell biology. Nucleic Acids Res. 46, 8038–8056 10.1093/nar/gky735 30124962 PMC6144788

[51] Wright E.P., Huppert J.L. and Waller Z.A.E. (2017) Identification of multiple genomic DNA sequences which form i-motif structures at neutral pH. Nucleic Acids Res. 45, 2951–2959 10.1093/nar/gkx090 28180276 PMC5605235

[52] Fojtik P., Kejnovska I. and Vorlickova M. (2004) The guanine-rich fragile X chromosome repeats are reluctant to form tetraplexes. Nucleic Acids Res. 32, 298–306 10.1093/nar/gkh179 14718550 PMC373289

[53] Renciuk D., Zemanek M., Kejnovska I. and Vorlickova M. (2009) Quadruplex-forming properties of FRAXA (CGG) repeats interrupted by (AGG) triplets. Biochimie 91, 416–422 10.1016/j.biochi.2008.10.012 19028545

[54] Schmidt M.H. and Pearson C.E. (2016) Disease-associated repeat instability and mismatch repair. DNA Repair (Amst.) 38, 117–126 10.1016/j.dnarep.2015.11.008 26774442

[55] Freudenreich C.H. (2018) R-loops: targets for nuclease cleavage and repeat instability. Curr. Genet. 64, 789–794 10.1007/s00294-018-0806-z 29327083 PMC6039234

[56] Kumari D., Lokanga R., Yudkin D., Zhao X.N. and Usdin K. (2012) Chromatin changes in the development and pathology of the Fragile X-associated disorders and Friedreich ataxia. Biochim. Biophys. Acta 1819, 802–810 10.1016/j.bbagrm.2011.12.009 22245581 PMC3370136

[57] Wang G. and Vasquez K.M. (2014) Impact of alternative DNA structures on DNA damage, DNA repair, and genetic instability. DNA Repair (Amst.) 19, 143–151 10.1016/j.dnarep.2014.03.017 24767258 PMC4216180

[58] Bacolla A. and Wells R.D. (2009) Non-B DNA conformations as determinants of mutagenesis and human disease. Molecular Carcinog. 48, 273–285 10.1002/mc.20507 19306308

[59] Rocha E.P. (2008) The organization of the bacterial genome. Annu. Rev. Genet. 42, 211–233 10.1146/annurev.genet.42.110807.091653 18605898

[60] West B.J., Allegrini P., Buiatti M. and Grigolini P. (2000) Non-normal statistics of DNA sequences of prokaryotes. J. Biol. Phys. 26, 17–25 10.1023/A:1005284418550 23345709 PMC3456183

[61] Jansen R., Embden J.D., Gaastra W. and Schouls L.M. (2002) Identification of genes that are associated with DNA repeats in prokaryotes. Mol. Microbiol. 43, 1565–1575 10.1046/j.1365-2958.2002.02839.x 11952905

[62] Kolsto A.B. (1997) Dynamic bacterial genome organization. Mol. Microbiol. 24, 241–248 10.1046/j.1365-2958.1997.3501715.x 9159511

[63] Cechova J., Lysek J., Bartas M. and Brazda V. (2017) Complex analyses of inverted repeats in mitochondrial genomes revealed their importance and variability. Bioinformatics 34, 1081–1085 10.1093/bioinformatics/btx729 PMC6030915 29126205

[64] Brazda V., Lysek J., Bartas M. and Fojta M. (2018) Complex analyses of short inverted repeats in all sequenced chloroplast DNAs. Biomed. Res. Int. 2018, 1097018 10.1155/2018/1097018 30140690 PMC6081594

[65] Horwitz M.S. and Loeb L.A. (1988) An E. coli promoter that regulates transcription by DNA superhelix-induced cruciform extrusion. Science 241, 703–705 10.1126/science.2456617 2456617

[66] Holder I.T., Wagner S., Xiong P., Sinn M., Frickey T., Meyer A. et al. (2015) Intrastrand triplex DNA repeats in bacteria: a source of genomic instability. Nucleic Acids Res. 43, 10126–10142 10.1093/nar/gkv1017 26450966 PMC4666352

[67] Bacolla A., Wang G. and Vasquez K.M. (2015) New perspectives on DNA and RNA triplexes as effectors of biological activity. PLoS Genet. 11, e1005696 10.1371/journal.pgen.1005696 26700634 PMC4689454

[68] Kikin O., D'Antonio L. and Bagga P.S. (2006) QGRS mapper: a web-based server for predicting G-quadruplexes in nucleotide sequences. Nucleic Acids Res. 34, W676–W682 10.1093/nar/gkl253 16845096 PMC1538864

[69] Brazda V., Kolomaznik J., Lysek J., Bartas M., Fojta M., Stastny J. et al. (2019) G4hunter web application: a web server for G-quadruplex prediction. Bioinformatics 35, 3493–3495 10.1093/bioinformatics/btz087 30721922 PMC6748775

[70] Yadav V.K., Abraham J.K., Mani P., Kulshrestha R. and Chowdhury S. (2008) Quadbase: genome-wide database of G4 DNA-occurrence and conservation in human, chimpanzee, mouse and rat promoters and 146 microbes. Nucleic Acids Res. 36, D381–D385 10.1093/nar/gkm781 17962308 PMC2238983

[71] Dhapola P. and Chowdhury S. (2016) Quadbase2: web server for multiplexed guanine quadruplex mining and visualization. Nucleic Acids Res. 44, W277–W283 10.1093/nar/gkw425 27185890 PMC4987949

[72] Rawal P., Kummarasetti V.B., Ravindran J., Kumar N., Halder K., Sharma R. et al. (2006) Genome-wide prediction of G4 DNA as regulatory motifs: role in *Escherichia coli* global regulation. Genome Res. 16, 644–655 10.1101/gr.4508806 16651665 PMC1457047

[73] Brazda V., Haronikova L., Liao J.C. and Fojta M. (2014) DNA and RNA quadruplex-binding proteins. Int. J. Mol. Sci. 15, 17493–17517 10.3390/ijms151017493 25268620 PMC4227175

[74] Day H.A., Pavlou P. and Waller Z.A. (2014) i-Motif DNA: structure, stability and targeting with ligands. Bioorg. Med. Chem. 22, 4407–4418 10.1016/j.bmc.2014.05.047 24957878

[75] Harris L.M. and Merrick C.J. (2015) G-quadruplexes in pathogens: a common route to virulence control? PLoS Pathog. 11, e1004562 10.1371/journal.ppat.1004562 25654363 PMC4412290

[76] Rhodes D. and Lipps H.J. (2015) G-quadruplexes and their regulatory roles in biology. Nucleic Acids Res. 43, 8627–8637 10.1093/nar/gkv862 26350216 PMC4605312

[77] Saranathan N. and Vivekanandan P. (2019) G-quadruplexes: more than just a kink in microbial genomes. Trends Microbiol. 27, 148–163 10.1016/j.tim.2018.08.011 30224157 PMC7127049

[78] Day H.A., Wright E.P., MacDonald C.J., Gates A.J. and Waller Z.A. (2015) Reversible DNA i-motif to hairpin switching induced by copper(II) cations. Chem. Commun. (Camb.) 51, 14099–14102 10.1039/C5CC05111H 26252811 PMC4563791

[79] Waller Z.A., Pinchbeck B.J., Buguth B.S., Meadows T.G., Richardson D.J. and Gates A.J. (2016) Control of bacterial nitrate assimilation by stabilization of G-quadruplex DNA. Chem. Commun. (Camb.) 52, 13511–4 10.1039/C6CC06057A 27805200 PMC5123632

[80] Abdelhamid M.A.S., Gates A.J. and Waller Z.A.E. (2019) Destabilization of i-Motif DNA at neutral pH by G-quadruplex ligands. Biochemistry 58, 245–249 10.1021/acs.biochem.8b00968 30350580

[81] Pinchbeck B.J., Soriano-Laguna M.J., Sullivan M.J., Luque-Almagro V.M., Rowley G., Ferguson S.J. et al. (2019) A dual functional redox enzyme maturation protein for respiratory and assimilatory nitrate reductases in bacteria. Mol. Microbiol. 111, 1592–1603 10.1111/mmi.14239 30875449 PMC6618116

[82] Iyer R.R., Pluciennik A., Napierala M. and Wells R.D. (2015) DNA triplet repeat expansion and mismatch repair. Annu. Rev. Biochem. 84, 199–226 10.1146/annurev-biochem-060614-034010 25580529 PMC4845744

[83] Chatterjee N. and Walker G.C. (2017) Mechanisms of DNA damage, repair, and mutagenesis. Environ. Mol. Mutagen. 58, 235–263 10.1002/em.22087 28485537 PMC5474181

[84] Shah K.A. and Mirkin S.M. (2015) The hidden side of unstable DNA repeats: mutagenesis at a distance. DNA Repair (Amst.) 32, 106–112 10.1016/j.dnarep.2015.04.020 25956860 PMC4522329

[85] Lahue R.S. and Slater D.L. (2003) DNA repair and trinucleotide repeat instability. Front. Biosci. 8, s653–s665 10.2741/1107 12700078

[86] Zhao X.N. and Usdin K. (2015) The repeat expansion diseases: the dark side of DNA repair. DNA Repair (Amst.) 32, 96–105 10.1016/j.dnarep.2015.04.019 26002199 PMC4522390

[87] Bowater R.P. and Wells R.D. (2001) The intrinsically unstable life of DNA triplet repeats associated with human hereditary disorders. Prog. Nucleic Acids Res. Mol. Biol. 66, 159–202 10.1016/S0079-6603(00)66029-4 11051764

[88] Krasilnikova M., Samadashwily G.M., Krasilnikov A.S. and Mirkin S.M. (1998) Transcription through a simple DNA repeat blocks replication elongation. EMBO J. 17, 5095–5102 10.1093/emboj/17.17.5095 9724645 PMC1170837

[89] Bowater R.P., Jaworski A., Larson J.E., Parniewski P. and Wells R.D. (1997) Transcription increases the deletion frequency of long CTG•CAG triplet repeats from plasmids in *Escherichia coli*. Nucleic Acids Res. 25, 2861–2868 10.1093/nar/25.14.2861 9207036 PMC146811

[90] Lin Y., Dent S.Y., Wilson J.H., Wells R.D. and Napierala M. (2010) R loops stimulate genetic instability of CTG.CAG repeats. Proc. Natl Acad. Sci. U.S.A. 107, 692–697 10.1073/pnas.0909740107 20080737 PMC2818888

[91] Reddy K., Tam M., Bowater R.P., Barber M., Tomlinson M., Nichol Edamura K. et al. (2011) Determinants of R-loop formation at convergent bidirectionally transcribed trinucleotide repeats. Nucleic Acids Res. 39, 1749–1762 10.1093/nar/gkq935 21051337 PMC3061079

[92] Zhao J., Bacolla A., Wang G. and Vasquez K.M. (2010) Non-B DNA structure-induced genetic instability and evolution. Cell. Mol. Life Sci. 67, 43–62 10.1007/s00018-009-0131-2 19727556 PMC3017512

[93] Hoeijmakers J.H. (2001) Genome maintenance mechanisms for preventing cancer. Nature 411, 366–374 10.1038/35077232 11357144

[94] Friedberg E.C. (2003) DNA damage and repair. Nature 421, 436–440 10.1038/nature01408 12540918

[95] Gorna A.E., Bowater R.P. and Dziadek J. (2010) DNA repair systems and the pathogenesis of *Mycobacterium tuberculosis*: varying activities at different stages of infection. Clin Sci (Lond) 119, 187–202 10.1042/CS20100041 20522025

[96] van der Veen S. and Tang C.M. (2015) The BER necessities: the repair of DNA damage in human-adapted bacterial pathogens. Nat. Rev. Microbiol. 13, 83–94 10.1038/nrmicro3391 25578955

[97] Uphoff S. and Sherratt D.J. (2017) Single-molecule analysis of bacterial DNA repair and mutagenesis. Annu. Rev. Biophys. 46, 411–432 10.1146/annurev-biophys-070816-034106 28375733

[98] Loeb K.R. and Loeb L.A. (1999) Genetic instability and the mutator phenotype. Am. J. Pathol. 154, 1621–1626 10.1016/S0002-9440(10)65415-6 10362784 PMC1866616

[99] Bacolla A., Wojciechowska M., Kosmider B., Larson J.E. and Wells R.D. (2006) The involvement of non-B DNA structures in gross chromosomal rearrangements. DNA Repair (Amst.) 5, 1161–1170 10.1016/j.dnarep.2006.05.032 16807140

[100] Wojcik E.A., Brzostek A., Bacolla A., Mackiewicz P., Vasquez K.M., Korycka-Machala M. et al. (2012) Direct and inverted repeats elicit genetic instability by both exploiting and eluding DNA double-strand break repair systems in mycobacteria. PLoS One 7, e51064 10.1371/journal.pone.0051064 23251422 PMC3519483

[101] Mendoza O., Bourdoncle A., Boule J.B., Brosh R.M. Jr. and Mergny J.L. (2016) G-quadruplexes and helicases. Nucleic Acids Res. 44, 1989–2006 10.1093/nar/gkw079 26883636 PMC4797304

[102] Shen J.C. and Loeb L.A. (2000) The Werner syndrome gene: the molecular basis of RecQ helicase-deficiency diseases. Trends Genet. 16, 213–220 10.1016/S0168-9525(99)01970-8 10782115

[103] Wu X. and Maizels N. (2001) Substrate-specific inhibition of RecQ helicase. Nucleic Acids Res. 29, 1765–1771 10.1093/nar/29.8.1765 11292849 PMC31322

[104] Moxon R., Bayliss C. and Hood D. (2006) Bacterial contingency loci: the role of simple sequence DNA repeats in bacterial adaptation. Annu. Rev. Genet. 40, 307–333 10.1146/annurev.genet.40.110405.090442 17094739

[105] Kashi Y., King D. and Soller M. (1997) Simple sequence repeats as a source of quantitative genetic variation. Trends Genet. 13, 74–78 10.1016/S0168-9525(97)01008-1 9055609

[106] Karlin S., Campbell A.M. and Mrazek J. (1998) Comparative DNA analysis across diverse genomes. Annu. Rev. Genet. 32, 185–225 10.1146/annurev.genet.32.1.185 9928479

[107] Groisman E.A. and Casadesus J. (2005) The origin and evolution of human pathogens. Mol. Microbiol. 56, 1–7 10.1111/j.1365-2958.2005.04564.x 15773974

[108] Power P.M., Sweetman W.A., Gallacher N.J., Woodhall M.R., Kumar G.A., Moxon E.R. et al. (2009) Simple sequence repeats in *Haemophilus influenzae*. Infect. Genet. Evol. 9, 216–228 10.1016/j.meegid.2008.11.006 19095084 PMC2651432

[109] Zhou K., Aertsen A. and Michiels C.W. (2014) The role of variable DNA tandem repeats in bacterial adaptation. FEMS Microbiol. Rev. 38, 119–141 10.1111/1574-6976.12036 23927439

[110] Peak I.R., Jennings M.P., Hood D.W. and Moxon E.R. (1999) Tetranucleotide repeats identify novel virulence determinant homologues in *Neisseria meningitidis*. Microb. Pathog. 26, 13–23 10.1006/mpat.1998.0243 9973577

[111] Hood D.W., Deadman M.E., Jennings M.P., Bisercic M., Fleischmann R.D., Venter J.C. et al. (1996) DNA repeats identify novel virulence genes in *Haemophilus influenzae*. Proc. Natl Acad. Sci. U.S.A. 93, 11121–11215 10.1073/pnas.93.20.11121 8855319 PMC38294

